# Supplementary material for: Swedish high-school pupils’ attitudes towards drugs in relation to drug usage, impulsiveness and other risk factors
Source: PeerJ. 2014 Jun 5;2:e410. doi: 10.7717/peerj.410 (PMC4060018; doi:10.7717/peerj.410)
Supplement: Table S1 [file peerj-02-410-s001.docx]

Table S1. Respondent’s characteristics.

| Variable | | *N* | *Mean* | *SD* | *t*(*df*) | *p* 2-tailed | *N*  Items | *Cronbach’s*  *alpha* |
| --- | --- | --- | --- | --- | --- | --- | --- | --- |
| **Age (years)** |  | 145 | 18.08 | 0.61 |  |  |  |  |
|  | Female | 74 | 18.03 | 0.57 |  |  |  |  |
|  | Male | 69 | 18.12 | 0.65 |  |  |  |  |
| **Barrat’s Impulsiveness Scale (BIS-11)** |  | 101 | 60.11 | 10.18 |  |  | 29 | 0.84 |
|  | Female | 48 | 61.98 | 10.53 |  |  |  |  |
|  | Male | 53 | 58.25 | 9.63 |  |  |  |  |
| **Did use drugs** |  | 46 | 59.62 | 10.23 | 3.99(144) | 0.000 |  |  |
| **Did not use drugs** |  | 100 | 67.04 | 10.88 |  |  |  |  |
| **Cognitive impulsiveness** |  | 146 | 2.13 | 0.53 |  |  |  |  |
|  | Female | 75 | 2.16 | 0.60 |  |  |  |  |
|  | Male | 69 | 2.11 | 0.45 |  |  |  |  |
| **Did use drugs** |  | 46 | 2.03 | 0.49 | 3.68(144) | 0.000 |  |  |
| **Did not use drugs** |  | 100 | 2.31 | 0.56 |  |  |  |  |
| **Risk factors total sum of scores** |  | 146 | 65.90 | 10.47 |  |  | 32 | 0.83 |
|  | Female | 62 | 63.95 | 9.97 | 1.95(121) | 0.053 |  |  |
|  | Male | 61 | 67.61 | 10.77 |  |  |  |  |
| **Did use drugs** |  | 43 | 71.53 | 10.20 | 4.59(123) | 0.000 |  |  |
| **Did not use drugs** |  | 82 | 62.95 | 9.39 |  |  |  |  |
| **Community as a risk factor for impulsiveness** |  | 141 | 21.94 | 3.96 |  |  | 11 | 0.72 |
|  | Female | 72 | 21.88 | 4.17 |  |  |  |  |
|  | Male | 67 | 21.96 | 3.75 |  |  |  |  |
| **Family as a risk factor for impulsiveness** |  | 137 | 47.36 | 10.01 |  |  | 24 | 0.88 |
|  | Female | 71 | 47.37 | 10.62 |  |  |  |  |
|  | Male | 64 | 46.73 | 8.84 |  |  |  |  |
| **Individual risk. Sensation-seeking. for impulsiveness** |  | 144 | 8.08 | 2.39 |  |  | 5 | 0.75 |
|  | Female | 75 | 7.72 | 1.88 |  |  |  |  |
|  | Male | 69 | 8.33 | 2.86 |  |  |  |  |
| **Attitude total sum of scores** |  | 143 | 81.22 | 13.42 |  |  | 21 | 0.72 |
|  | Female | 74 | 82.54 | 14.98 |  |  |  |  |
|  | Male | 67 | 79.96 | 11.50 |  |  |  |  |
| **Drug Abuse total sum of scores** |  | 45 | 36.84 | 3.45 |  |  | 15 | 0.81 |
|  | Female | 15 | 36.67 | 3.46 |  |  |  |  |
|  | Male | 28 | 37.04 | 3.45 |  |  |  |  |
| **Protective factors total sum of scores** |  | 137 | 30.26 | 6.80 |  |  | 14 | 0.84 |
|  | Female | 72 | 29.47 | 6.93 |  |  |  |  |
|  | Male | 63 | 30.78 | 6.35 |  |  |  |  |
| **Did use drugs** |  | 45 | 32.04 | 7.15 | 2.19(135) | 0.031 |  |  |
| **Did not use drugs** |  | 92 | 29.38 | 6.47 |  |  |  |  |
| **Protective factor Family total sum of scores** |  | 140 | 13.82 | 4.43 |  |  | 7 | 0.86 |
|  | Female | 74 | 13.96 | 4.68 |  |  |  |  |
|  | Male | 66 | 13.47 | 4.05 |  |  |  |  |
| **Protective factor Community total sum of scores** |  | 143 | 9.01 | 2.58 |  |  | 3 | 0.88 |
|  | Female | 74 | 8.92 | 2.81 |  |  |  |  |
|  | Male | 67 | 9.06 | 2.34 |  |  |  |  |
